# Visual outcome of laser treatment in diabetic macular edema: Study from an Urban Diabetes Care Center

**DOI:** 10.12669/pjms.325.10597

**Published:** 2016

**Authors:** Rashid Alvi, Muhammad Saleh Memon, Samad Shera, Seema N. Mumtaz, Sikander Ali Shaikh, Muhammad Faisal Fahim

**Affiliations:** 1 Rashid Alvi, Department of Ophthalmology, Diabetic Association of Pakistan (DAP), WHO Collaborating Center, Karachi, Pakistan; 2 Muhammad Saleh Memon, Isra Ophthalmic Research & Development Center, Al-Ibrahim Eye Hospital, Isra postgraduate Institute of Ophthalmology, Karachi, Pakistan; 3 Samad Shera, Department of Ophthalmology, Diabetic Association of Pakistan (DAP), WHO Collaborating Center, Karachi, Pakistan; 4 Seema N. Mumtaz, Isra Ophthalmic Research & Development Center, Al-Ibrahim Eye Hospital, Isra postgraduate Institute of Ophthalmology, Karachi, Pakistan; 5 Sikander Ali Shaikh, Isra Ophthalmic Research & Development Center, Al-Ibrahim Eye Hospital, Isra postgraduate Institute of Ophthalmology, Karachi, Pakistan; 6 Muhammad Faisal Fahim, Isra Ophthalmic Research & Development Center, Al-Ibrahim Eye Hospital, Isra postgraduate Institute of Ophthalmology, Karachi, Pakistan

**Keywords:** Diabetic retinopathy, Clinically significant macular edema, Grid laser, Focal laser

## Abstract

**Objective:**

To determine the visual outcome of laser treatment in clinically significant macular edema.

**Methods:**

This interventional and qausi experimental study was carried out at Diabetic Association of Pakistan (DAP) during January 2011 and December 2012. Approval was taken from Research Ethical Committee of Isra Postgraduate Institute of Ophthalmology. Records of 925 eyes of 464 patients with “Clinical Significant macular edema” (CSME), treated with laser photocoagulation were analyzed. Best-corrected visual acuity (BCVA) at the time of presentation and at the last follow up, minimum of one year and maximum of 45 months was recorded and compared. SPSS version 20.0 was used to analyze the data.

**Results:**

Diabetic retinopathy was found in 20.3% (1777) of 8742 diabetic attending DAP Hospital” amongst whom 39.6% (705) had Sight threatening diabetic retinopathy. Laser was advised in 96.4% (680) individuals, accepted by 70.5% (480) individuals. Amongst 960 eyes of 480 patients who accepted laser, 925 eyes had clinically significant macular edema and 35 eyes had PDR who are not included in this study. Amongst 925 eyes with CSME, Grid laser was done in 913 eyes (99%) and focal laser was done in 12 eyes (1%). After a follow up of 12 to 45 months, it was found that best corrected visual acuity had declined in 2.4% (22) eyes, stabilized in 67% (619) eyes and improved in 30.7% (284) eyes. One line improvement on Snellen’s chart was fond in 21.3% (197) eyes, 2 lines in 8% (74) eyes, 3 lines in 1.2% (12) eyes and 4 lines in one (0.1%) eye with p-value of 0.000.

**Conclusion:**

Laser therapy is an effective treatment in stabilizing/improving the vision in diabetic macular edema particularly at those centers where only Argon Laser is available and OCF, FFA facilities do not exist.

## INTRODUCTION

Diabetic retinopathy (DR) is leading cause of blindness in the working-age population of developing countries.^1^ In Pakistan, every fourth diabetic patient has diabetic retinopathy and 40% of DR have sight threatening diabetic retinopathy (STDR).^2^,^3^ Improved control of diabetes can prevent DR, slow the progression of retinopathy.^4^ Once patient develops STDR, intervention is indicated. Controlled trials have shown that pan retinal photocoagulation (PDR) in proliferative diabetic retinopathy (PDR) and grid laser/focal photocoagulation in “Clinically significant macular edema” (CSME) are effective in reducing vision loss by 50% or more.^5^-^7^

In spite of its effectiveness, laser is a destructive process and causes constriction of visual fields, scotomas and decreased contrast sensitivity. Introduction of intravitreal anti-vascular endothelial growth factors (anti-VEGF) have opened up a new venue for of treatment of CSME and PDR^8^-^11^ and is replacing laser therapy as a first line of treatment especially in CSME.^12^,^13^ With all its benefits, Anti- VEGFs have their limitations like availability, need for repeated injection, cost, safety and strict monitoring.

In comparison, Laser therapy is available in 85% of the public health sector hospitals,^14^ is relatively cheaper and strict monitoring is not essential. This makes laser application still the treatment of choice in CSME and PDR when affordability and follow up is always a problem. Several national studies have shown effectivity of laser therapy in PDR.^15^-^17^ This study, intends to see effectivity of laser therapy in CSME.

## METHODS

This was an interventional and qausi experimental study carried out at Diabetic Association of Pakistan (DAP), a World Health Organization (WHO) collaborating center in Karachi, from January 2011 to December 2012. An eye clinic was established at DAP hospital with necessary equipment and human resource for diabetic retinopathy (DR) screening and laser therapy. Research Ethical Committee (REC) of Isra Post Graduate institute of ophthalmology gave ethical approval for the study.

All the new patients registered at DAP, irrespective of cast, socio economic conditions, religion and gender were referred to eye clinic for retinal screening and management of DR. Informed consent, data on demographic and clinical parameters was collected from each patient on a pre-tested Performa. Best- corrected visual acuity of each patient was recorded and entered in the Performa by an optometrist. All the patients were then referred for fundus photography.

### Image Acquisition

Retinal screening was done with a non-Mydriatic fundus camera Canon CR-1 by the optometrist trained in fundus photography. Screening was performed without dilatation of the pupil. Two 45° retinal images, one center to the optic disc and other center to the macula of each eye were taken and stored by patient’s name and identification number on the hard disk and a compact disc (CD). The images acquired were read by the optometrist, trained in fundus photography. Patients with normal fundus were advised follow-up and excluded from the study. Patients with any sign of retinopathy or inconclusive photographs were referred to ophthalmologist trained in medical retina at eye clinic of DAP.

### Grading of retinopathy

Each patient had full ophthalmic assessment, which included slit-lamp bio-microscopy with 90-D fundus lens and binocular indirect ophthalmoscopy after fully dilating the pupil fully with Tropicamide 1%. If the fundi were difficult to assess even after dilatation of pupil, patients were referred to an eye center for management. Grading of diabetic retinopathy was done according to modified Airlie House Classification, adopted and modified by Early Treatment Research Group.^18^ Diabetic retinopathy was classified with regard to severity, into non-proliferative Diabetic retinopathy (NPDR), proliferative Diabetic retinopathy (PDR), macular edema alone or with NPDR/PDR or Advanced Diabetic Eye Diseases (ADED). Mild, moderate and severe NPDR without CSME were categorized as Non Sight Threatening DR (NSTDR) group and advised follow-up. PDR, CSME alone or in combination other types of DR were included in the category of STDR and advised Laser therapy. Patients with Advanced Diabetic Eye Diseases (ADED) were referred for pars-plana vitrectomy.

### Laser application

All the patients signed an informed consent written in local language. Socio-demographic Data was entered on a prescribed form including age, gender, address, contact number, occupation and level of education. Pupil was fully dilated. One consultant (RA) did all the lasers, using Double frequency YAG laser (S 32 Taiwan) with Pan Funduscopic lens. The patients with CSME alone or associated with any other type of diabetic retinopathy were included in the study. The patients were treated with grid pattern or focal laser in one session. For grid laser, burns used were of spot size 50-100 µm and 0.05-0.1 second’s duration. Burns were applied to macular area of diffuse retinal thickening, treating no closer than 500 µm from the foveola and 500 µm from the optic disc. Power was adjusted to give mild reaction. For focal laser, 75-100 µm spot size was used with duration of and 0.05-0.1 seconds. Burns were placed 500=3000 µm from foveola.

### Follow-up

The patients were advised follow up at monthly and later on quarterly intervals till one year. On each visit, best- corrected visual acuity (BCVA) was recorded and fundus photograph taken.

### Statistical analysis

Data was analyzed by Statistical package for Social Sciences (SPSS) version 20.0. The entire continuous variables were presented as mean ± standard deviation and categorical variables were expressed as frequency and percentage. Chi square test was applied to see the significance. P-value less than 0.05 considered statistically significant.

## RESULTS

Retinal screening was done in 8742 diabetics with the Median age of 53 years (range 19-102) and female to male ratio of 1: 0.53 (5708 females and 3034 males). Type-II diabetes was present in 92.5% (8083) patients, type-1 was present in 6.4% (562) patients, and 1.1% (97) patients had gestational diabetes mellitus. As regards the duration of diabetes, 46.6% (4070) respondents had diabetes for 1-5 years, 26.4% (2309) had diabetes for 6-10 years and 27% (2363) respondents had diabetes for more than 10 years. When the duration of diabetes was related to CSME, it was found that 30.3% had the disease for less than five years, 23% had disease for 6-10 years and 46.5% had diabetes with more than 10 year. Significant association was found between duration of diabetes and presence of CSME (P<0.003). (Table-I)

**Table-I:** Demography Patients with Diabetes.

*Characteristics (n= 8742)*	*% (frequency)*
Age (Median, Years)	53 (19-102)
Male	34.7% (3034)
Female	65.3% (5708)
*Type of Diabetes (n= 8742)*	
Type I	6.4 % (562)
Type II	92.5% (8083)
GDM	1.1% (97)
*Duration of Diabetes (n= 8742)*	
1-5 Years	46.6% (4070)
6-10 years	26.4% (2309)
> 10 years	27.5% (2363)
*Duration of Diabetes in patients with CSME (n=464)*	
1-5 Years	30.3% (141)
	3.4% of all diabetic
6-10 years	23% (107)
	4.6% of all diabetics
> 10 years	46.5% (216)
	9.1% of all diabetics

*Data presented as Median (Range), Percentage & (frequency)

Diabetic retinopathy was found in 20.3% (1777) individuals amongst whom 39.6% (705) had STDR. Laser was advised in 96.45% (680) patients and accepted by 70.5% (480) amongst whom 464 (925 eyes) respondents had CSME. Association of CSME with other types of DR is shown in (Table-II).

**Table-II:** Showing association of CSME with other types of DR (n= 464).

*Diagnosis*	*Frequency (%)*
CSME alone	10.7% (50)
CSME + Mild NPDR	82.9% (385)
CSME +Moderate NPDR	3.45% (16)
CSME +PDR	2.8% (13)

Total	464

*Data presented as Percentage & (frequency)

Grid laser was done in 913 eyes and Focal laser was done in 12 eyes. Best-corrected visual acuity on Snellen’s chart at the last visit had deteriorated in 2.4% (22) eyes, stabilized in 67% (619) eyes and improved in 30.6% (284) eyes. One line improvement on Snellen’s chart was fond in 21.3% (197) eyes, 2 lines in 8% (74) eyes, 3 lines in 1.2% (12) eyes and 4 lines in 0.1% (01) eye. (Table-III).

**Table-III:** Status of vision in eyes with CSME after laser application (n= 925 eyes).

*Status of Vision*	*% (Frequency)*
Deterioration of vision	2.3% (22)
Stable vision	66.9 % (619)
Improvement in vision	30.7% (284)
Total	925

*Frequency of Improvement n=284 eyes*	

1 line improvement	21.2% (197 eyes)
2 line improvement	8.0 % (74 eyes)
3 line improvement	1.29 % (12 eyes)
4 line improvement	0.1 % (01 eyes)

*Data presented as Percentage & (frequency)

Follow up pattern was not according to the study protocol (monthly and quarterly). It ranged widely from 12 to 45 months (January 2011 to March 2015). Out of total 464 cases, 43.7% (203) attended once, 42.6% (198) twice, 4.5% (21) thrice, and 6.8% (32) four times and 2.1% (10) attended 5 times. Last visit of each patient was different in time.

## DISCUSSION

Laser photocoagulation became the standard treatment for diabetic macular edema after publication of results from the Early Treatment Diabetic Retinopathy Study in 1990. The ETDR results demonstrated that, focal photocoagulation, grid laser or both were effective in reducing the risk of moderate visual loss due to diabetic macular edema.^19^ However, macular edema results in thickening of retina, which acts like prism and spreads out the light resulting in larger and diffuse burn and unpredictable results. This prompted researchers to look for alternate treatment modalities. Intra vitriol triamcinolone was a promising drug; but was associated with glaucoma and cataract formation.^20^ Anti-VEGFs have proved effective, claiming better results.^21^-^23^ Bolt studies have supported the use of bevacizumab (Avastin; Genentech, San Francisco, CA, USA) as compared to Laser in patients with center-involving CSME without advanced macular ischemia.^24^ It is to be noted that in anti-VEGF treatment, regular visits, monitoring and repeated injections are necessary. After laser photocoagulation, follow up for continued care is not as important as after anti-VEGFs. Moreover, monthly injections^25^ makes it unaffordable for most of the patients. Even the cheapest ant-VEGF, Bevacizumab is not affordable and accessible to the patients in semi-urban and rural areas.

This study shows that laser therapy is quite effective in preserving vision in DME. Best- corrected visual acuity (BCVA) on Snellen’s chart had remained stabilized in 65.8%, improved in 31.7% with improvement by one line in 22% (210 eyes), two lines in 8.56% (82 eyes), three lines in 1.25% (2 eyes) and four lines in 0.1% (one eye). Visual acuity had deteriorated only in 2.3% (22) eyes. These findings are quite comparable to international studies.^26^,^27^ National studies have shown favorable results of laser therapy in PDR. Chandka study reported improvement in 39.6% (143) eyes, deterioration in 50% (183).^15^ Dow Medical University study reported visual acuity improvement in 40.2% (147) eyes, deterioration in 36.9% (183).^16^ Study from Armed Forces Institute of ophthalmology Rawalpindi^17^ concluded that Laser PRP with Pattern scan laser is safe and effective in patients with combined presentation of PDR and DME. Present study has shown that laser therapy alone is quite effective in CSME.

There are few confounders in this study. Parameter of diabetes control was fasting (109 mg/l) and post-prandial glucose level (140-190 mg/l). Lipid profile and HB A1C was done in affording patients only. Second confounder was absence of OCT and FFA as diagnostic and monitoring tool. The diagnosis of CSME was totally on clinical examination. OCT and FFA was not available in this center. These facilities were neither affordable nor accessible for most of the patients. All the patients were recommended OCT and who agreed for investigations were referred to a tertiary center (Al Ibrahim Eye Hospital) and excluded from the study. Aim was to do the study in a set up comparable to District headquarter hospital of rural setup where the ophthalmologist has to depend upon clinical skills only. We considered visual results as a sign of successful treatment and not the anatomical success shown by OCT because of its non-availability.

Final confounder may be the pattern of follow up of the patients. It was observed that follow up was not according to the study protocol (monthly and quarterly) but according to patient’s convenience. Minimum follow up during this study was one years and maximum of 45 months. It was observed that almost all the patient attended the center one or more times. Out of total 464 macular edema patients of present study, 43.7% (203) attended once, 42.65 (198) attended twice, 4.5% (21) attended thrice, 6.8% (32) attended four times while 2.1% (10) came five times. This is an important indicator of our patient’s behavior showing importance of considering patient’s convenience in giving follow up date.

## CONCLUSION

Laser therapy is an effective treatment in stabilizing/improving the vision in diabetic macular edema particularly at those centers where only argon laser is available and facilities such as OCT, FFA do not exist. Future study is recommended to investigate the combination of laser photocoagulation with anti-vascular endothelial growth factors (anti-VEGFs).
